# Beneficial Effects of Rikkunshito, a Japanese Kampo Medicine, on Gastrointestinal Dysfunction and Anorexia in Combination with Western Drug: A Systematic Review

**DOI:** 10.1155/2014/519035

**Published:** 2014-03-20

**Authors:** Sachiko Mogami, Tomohisa Hattori

**Affiliations:** Tsumura Research Laboratories, Tsumura & Co., 3586 Yoshiwara, Ami-Machi, Inashiki-Gun, Ibaraki 300-1192, Japan

## Abstract

*Background.* Kampo medicines are traditional herbal medicines which have been approved for medicinal use by the Japanese Ministry of Health and Welfare and are currently being used more and more, often in combination with Western drugs. Thus, the need for investigation of interactions between Kampo medicines and Western drugs is now widely recognized. *Aim.* To summarize the effects and drug interactions of rikkunshito, a Kampo medicine often prescribed for upper gastrointestinal disorders and anorexia. *Methods.* Animal and human studies were systematically reviewed to identify published data on rikkunshito. Results describing its effects were abstracted, with an emphasis on drug interactions. *Results and Discussion.* Rikkunshito ameliorates anorexia induced by anticancer drugs, improves quality of life scores, and can even prolong survival compared with monotherapy. Rikkunshito combined with proton pump inhibitor therapy is shown to be useful in the treatment of PPI-refractory gastroesophageal reflux disease patients and patients with gastrointestinal symptoms after endoscopic submucosal dissection. Rikkunshito reduces antidepressant-induced adverse events and improves quality of life without influencing antidepressant effects. *Conclusions.* Rikkunshito shows ameliorative effects on adverse reactions induced by various Western drugs and can achieve better results (e.g., anticancer drugs and proton pump inhibitor) without influencing the efficacy and bioavailability of Western drugs.

## 1. Introduction

Herbal therapy has been used in Asia and other parts of the world for thousands of years and is currently manufactured in Japan as “Japanese Traditional (herbal) or Kampo medicines.” These medicines are standardized with regard to quality and quantity of its ingredients and have been approved by the Japanese Ministry of Health and Welfare. At present, almost 90% of physicians in Japan use Kampo medicines in daily practice, sometimes as the first choice. Because Kampo medicines are now being used more and more, often in combination with Western drugs, the need for investigation of interactions between Kampo medicines and Western drugs is now widely recognized. An emerging therapeutic target for Kampo medicines in clinical practice is gastrointestinal functional disorders, in which conventional pharmacotherapy is either only partly effective or associated with adverse events. Rikkunshito, a Kampo medicine, is often prescribed for upper gastrointestinal disorders such as functional dyspepsia (FD), gastroesophageal reflux, and anorexia [1–3] and is prepared by compounding eight of the following crude drugs:* Atractylodis lanceae rhizoma*,* Ginseng radix*,* Pinellia tuber*,* Hoelen*,* Zizyphi fructus*,* Aurantii nobilis pericarpium*,* Glycyrrhizae radix*, and* Zingiberis rhizome*. The present paper reviews the physiology and clinical benefits of rikkunshito, with special focus on rikkunshito-Western drug combination therapies.

## 2. Methods

A literature search was performed on PubMed using the keywords "rikkunshito," "rikkunshi-to," "TJ-43" (product ID), and "Liu-Jun-Zi-Tang" (Chinese pronunciation). After accumulating a combined list of studies, publications not examining the use of rikkunshito and those not written in English were excluded. Data abstraction was performed to characterize physiological and clinical effects and drug interactions of rikkunshito. Because a formal meta-analysis was not possible based on variations in study populations, disorders, and study protocols retrieved, data are presented descriptively with regard to the physiological and clinical effects of rikkunshito.

## 3. Results and Discussion

The initial search terms yielded 2 books, 14 reviews, and 66 original manuscripts related to rikkunshito, of which 30 were human studies and 36 were animal and* in vitro* studies. Of these 30 human studies, 10 concerned the use of Western drugs in combination with rikkunshito (Table 1); of the 36 animal and* in vitro* studies, 6 concerned the use of Western drugs in combination with rikkunshito (1 of these included a human study) and 1 investigated the effects of rikkunshito on human drug metabolic enzymes.

### 3.1. Clinical Applications of Rikkunshito

A double-blinded, randomized, placebo-controlled trial on rikkunshito was conducted by Tatsuta and Iishi in 42 patients with chronic idiopathic dyspepsia [15]. Gastric emptying was significantly accelerated and gastrointestinal symptoms were significantly reduced in patients treated with rikkunshito for 7 days, indicating that rikkunshito has a prokinetic action on gastric emptying and may be useful in treating FD. Arai et al. conducted a parallel, randomized controlled trial to investigate the effects of rikkunshito on upper gastrointestinal symptoms and ghrelin levels in 27 patients with FD treated with either rikkunshito or domperidone for 4 weeks [16]. Gastrointestinal Symptom Rating Scale (GSRS) questionnaire scores significantly improved in both groups but plasma ghrelin levels significantly increased only in the rikkunshito group and good correlation was found for improvements in reflux and indigestion symptoms. A study on the effects of rikkunshito on gastric accommodation reflex and duodenogastric motility was conducted in 16 patients with FD using extracorporeal ultrasonography before and after 14 days of treatment [17]. GSRS scores for abdominal pains, heartburn, and abdominal distension significantly decreased and the expansion rate of the proximal stomach was significantly greater after treatment. A study on the effects of 4-week treatment with rikkunshito in 19 patients with gastric cancer who had undergone proximal gastrectomy more than 6 months previously was conducted by Gunji et al. [18]. Patients' body weight significantly increased after the treatment course, and, in a subgroup analysis of patients showing a GSRS score of ≥2 before treatment, the mean total GSRS score significantly improved after treatment because of significant improvements in the subscale scores for abdominal pain, acid reflux, diarrhea, and constipation. Significant attenuation of post-gastrectomy gastrointestinal symptoms and increase in the ratio of acyl-/total ghrelin concentration by rikkunshito treatment for 4 weeks were also reported by Takiguchi et al. in 25 patients who had undergone distal gastrectomy or total gastrectomy [19]. Takahashi et al., in a crossover study, examined the clinical effects of rikkunshito in 11 patients who were to undergo pylorus-preserving gastrectomy for early gastric cancer [20]. Rikkunshito significantly improved emptying of solid meals from the remnant stomach according to (99 m)Tc-labeled solid scintigraphy, and stasis-related symptoms were significantly reduced during the treatment. Yagi et al. evaluated the effect of rikkunshito on symptoms and gastric myoelectric activity in 8 dyspeptic pediatric patients whose symptoms had persisted for over 1 year after gastrointestinal surgery. Following rikkunshito therapy, all patients exhibited symptomatic relief and a significant decrease in the mean symptom scores, along with increased gastric contractile activity according to electrogastrography [21]. Symptomatic relief and improved gastric emptying by rikkunshito administration were reported by Kawahara et al. in 9 handicapped patients using the ^13^C-acetate breath test and the BreathID system [22]. Kawahara et al. also investigated the effects of rikkunshito on clinical symptoms and esophageal acid exposure in 8 children with symptomatic gastroesophageal reflux disease (GERD) before and after 7 days of therapy [23]. Treatment relieved symptoms and reduced the distal esophageal acid exposure through improvement in esophageal acid clearance. The largest and most comprehensive clinical study was conducted by Harasawa et al. in 1998 in 235 patients with dysmotility-like dyspepsia although it is reported in Japanese and not found in the PubMed search but outlined in English elsewhere [24–26].

### 3.2. Effects of Rikkunshito in Nonclinical Studies

#### 3.2.1. Effects on Upper Gastrointestinal Function

The effects of rikkunshito on gastric adaptive relaxation have been reported in isolated guinea pig stomachs [27], conscious dogs [28], and isolated fundus smooth muscle from diabetic neuropathic rats with gastric dysmotility [29]. Rikkunshito not only increased gastric adaptive relaxation at the basal level but also ameliorated inhibited relaxation by the nitric oxide synthase inhibitor [27]. Improvement in gastric accommodation was also reported in virtual reality stress-imposed healthy humans [30]. Rikkunshito was reported to stimulate gastrointestinal contractions in the interdigestive state and gastric emptying in conscious dogs through cholinergic neurons and serotonin type-3 receptors [31]. The enhancement effect of rikkunshito was also reported in delayed gastric emptying induced by either nitric oxide synthase inhibitor (active components hesperidin (derived from* Aurantii nobilis pericarpium*) and L-arginine (derivation not determined)) [32] or serotonin [33]. Rikkunshito was reported to ameliorate the reduced voluntary movement in reflux esophagitis model rats and improve barrier function of the esophageal mucosa by restoring tight junction protein expression [34] and also reported to show great capacity to absorb bile salts [35]. Preventive effects of rikkunshito were also reported on gastric mucosal injury induced by either repeated electrical stimulation of the gastric artery or ethanol treatment via modulating platelet-activating factor production and oxidative granulocyte activation [36], in a nitric oxide-dependent manner [37] and by increasing surface mucin content [38].

#### 3.2.2. Effects on Ghrelin

Ghrelin, a 28-residue octanoylated peptide, is an endogenous ligand of the growth hormone secretagogue receptor (GHS-R) [39]. Ghrelin is known to play a role in both growth hormone release and stimulation of gastric motility and food intake [40–42]. Various studies are reported with regard to investigating the effects of rikkunshito on ghrelin secretion and signaling. Rikkunshito was reported to increase plasma ghrelin levels in humans and mice [43] and dogs [31] and was also reported to restore the decreased plasma ghrelin levels induced by serotonin release in rats. This rikkunshito effect is mediated by the serotonin 2B/2C receptor (5-HT_2B_R/5-HT_2C_R) antagonistic effect, the active components of rikkunshito including 3,3′,4′,5,6,7,8-heptamethoxyflavone and hesperidin (*Aurantii nobilis pericarpium*), and isoliquiritigenin (*Glycyrrhizae radix*) [44, 45]. Rikkunshito, hesperidin, and isoliquiritigenin ameliorated reduced hypothalamic ghrelin secretion and reduction in GHS-R signal transduction in the hypothalamus via 5-HT_2C_R antagonism [46, 47]. The potentiating effects on ghrelin signaling* in vitro* were also demonstrated in GHS-R-expressing cells, showing significantly sustained increase in intracellular Ca^2+^ levels induced by ghrelin, mediated by the increased binding ability of ghrelin to its receptor following pretreatment by rikkunshito or its one of the active components, atractylodin (*Atractylodis lanceae rhizoma*). The inhibitory effects on activities of ghrelin metabolizing enzymes, which inactivate ghrelin to deacylated form, were investigated by Sadakane et al. [48]. Several components of rikkunshito, such as glycycoumarin (*Glycyrrhizae radix*) and pachymic acid (*Hoelen*), were reported to show inhibitory activity against human ghrelin-deacylating enzyme, butyrylcholinesterase. In surgically induced GERD model rats, the ameliorative effects of exogenous ghrelin on reduced antral motility were observed only after pretreatment with rikkunshito, indicating that this medicine had restored gastrointestinal motility by reversing impaired ghrelin signaling [49].

#### 3.2.3. Effects on Other Hormones

Rikkunshito is reported to induce significant increases in plasma somatostatin and gastrin levels compared with a placebo group in healthy subjects but not in motilin, vasoactive intestinal peptide, calcitonin gene-related peptide, and substance P levels [50, 51]. It is also reported that rikkunshito suppresses increases in plasma adrenocorticotropic hormone levels, cortisol levels, and neuropeptide Y levels compared with the response to a placebo under stress conditions by repetitive blood sampling in health volunteers [52, 53].

#### 3.2.4. Effects in Anorexia Models

Rikkunshito and its components nobiletin (*Aurantii nobilis pericarpium*), heptamethoxyflavone, and isoliquiritigenin reportedly ameliorated compromised ghrelin reactivity in the hypothalamus and regulation of its secretion in mice with aging-associated anorexia via inhibition of phosphodiesterase type 3 [54]. These effects are considered to be mediated by suppression of leptin signaling in the hypothalamus by increased levels of cyclic adenosine monophosphate. Rikkunshito ameliorated reduced feeding behavior in an urocortin-1-induced anorexia model, and its effect was abolished by GHS-R antagonist coadministration [55]. Rikkunshito also attenuated anorexia induced by novel environmental change, by increasing plasma ghrelin levels via antagonism to 5-HT_1B_/_2C_R [56] and 5-HT_2B_R [57]. Effects of rikkunshito in a cancer anorexia-cachexia model were reported by Fujitsuka et al. [6], where amelioration in anorexia, gastrointestinal dysmotility, muscle wasting, and anxiety-related behavior as well as prolonged survival were recorded. These effects were mediated by rikkunshito's active components, hesperidin and atractylodin, which led to potentiated ghrelin secretion and receptor signaling, respectively. Ameliorative effects of rikkunshito on anorexia and body composition change is also reported in novel stomach cancer cachexia model by Terawaki et al. [58]. On the other hand, Tsubouchi et al. recently reported the protective effects of rikkunshito against acute lung injury by protecting the alveolar epithelial cells and regulating lung inflammation independently of the ghrelin system [59].

### 3.3. Rikkunshito and Western Drug Interactions

#### 3.3.1. Rikkunshito and Anticancer Drugs

Adverse reactions such as chemotherapy-induced nausea and vomiting often interfere with continuation of chemotherapy. The effects of rikkunshito on such adverse reactions were investigated in docetaxel/5-FU/cisplatin therapy, which is useful in the treatment of advanced esophageal cancer, as a prospective randomized study [4]. Nineteen patients who were to undergo docetaxel/5-FU/cisplatin therapy were randomly assigned to rikkunshito-treated and nontreated groups. Incidence of symptoms, nausea score, mood score, and activity of daily living score in quality of life (QOL) scoring were significantly lower in the rikkunshito-treated group than in the control group. The effects of rikkunshito on S-1/cisplatin chemotherapy-induced anorexia and ghrelin secretion were also investigated in a crossover design [5]. Ten unresectable or relapsed patients with gastric cancer were randomly divided between two groups. In the rikkunshito-on period, no cisplatin-induced decrease was observed for plasma ghrelin levels, average oral intake was significantly higher, and the grade of anorexia was significantly lower compared to those in the rikkunshito-off period. The effects of rikkunshito were also investigated as a retrospective study in 39 patients treated with gemcitabine who had pathologically proven stage III/IV pancreatic cancer with ascites. Median survival in patients treated with rikkunshito was significantly prolonged compared with that in patients treated with gemcitabine alone [6].

Combined administration of rikkunshito (1000 mg/kg) and cisplatin (2 or 4 mg/kg) was also reported in animal studies. In a cisplatin-induced anorexia model, rikkunshito was reported to ameliorate reduced food intake by reversing reduced plasma ghrelin levels, reduced hypothalamic ghrelin secretion, and decreased GHS-R signal transduction [44, 46–48]. It was also reported that combined administration of rikkunshito (500 mg/kg) and cisplatin (1 mg/kg) significantly prolonged survival in tumor-bearing rats compared with cisplatin monotherapy [6].

#### 3.3.2. Rikkunshito and Proton-Pump Inhibitors (PPI)

The relative efficacy of rikkunshito in combination with PPI, rabeprazole, and a double dose of rabeprazole was compared by Tominaga et al. in a prospective, multicenter, randomized, parallel comparative study in 104 PPI-refractory GERD patients [7]. After 4-week treatment with rabeprazole, patients were randomly assigned to either combination therapy (rikkunshito with a standard dose of rabeprazole) or a double dose of rabeprazole. Both treatment regimens significantly decreased the frequency scale for the symptoms of GERD in both groups. With regard to the therapeutic improvement rate, there were also significant effects in both groups. However, in the subgroup analysis based on reflux esophagitis/nonerosive GERD (NERD), the improvement rate for male patients with NERD in the rikkunshito group was significantly higher than that for male patients in the other group. These studies indicate that rikkunshito in combination with PPI therapy may be a useful new strategy for treatment of PPI-refractory patients. The effects of rikkunshito on laryngopharyngeal reflux (LPR) symptoms and gastric emptying in 22 patients with proton-pump inhibitor PPI-refractory LPR were investigated by Tokashiki et al. as a prospective, randomized, parallel comparative study [8]. Following 2 weeks of treatment with PPI monotherapy, patients were randomly divided between two treatment groups, rikkunshito monotherapy and rikkunshito plus lansoprazole. Following 4 weeks of treatment in both groups, the authors observed significantly decreased global sensation visual analog scale (VAS) scores, which showed significant positive correlation with improvement in gastric emptying. The VAS score for sore throat significantly decreased following treatment with rikkunshito plus PPI but not by rikkunshito alone. Effects of rikkunshito on gastrointestinal symptoms following endoscopic submucosal dissection (ESD) were evaluated in combination with rabeprazole by Uehara et al. in a prospective, randomized, parallel, comparative study [9]. Patients who were scored ≥3 more than the average GSRS score for abdominal pain or indigestion 6–8 days after ESD were randomized to either of the two groups (PPI monotreatment group, *n* = 5, or a PPI + rikkunshito group, *n* = 8). Overall GSRS score and abdominal pain score were significantly improved only in PPI plus rikkunshito group.

#### 3.3.3. Rikkunshito and Antidepressant Agents

Upper gastrointestinal symptoms such as nausea and vomiting are common adverse events associated with the administration of selective serotonin reuptake inhibitors (SSRIs) and may result in discontinuation of drug therapy in patients with a depressive disorder. A study on the effects of rikkunshito on gastrointestinal symptoms and antidepressant effects was performed by Oka et al. [10] in a randomized, controlled study of 50 patients with depressive disorder treated by fluvoxamine. Patients were divided into two groups, fluvoxamine and fluvoxamine plus rikkunshito, with administration over 8 weeks. The numbers of patients complaining of adverse events or nausea in the combination group were lower than those in the fluvoxamine group. GSRS scores improved in the combination group but not in the fluvoxamine group. Self-Rating Depression Scale scores were not different between the two groups at all assessment points.

Functional gastrointestinal symptoms are frequently found in elderly patients with dementia and treated by the administration of antidepressants or second-generation antipsychotics, but with the risk of side effects. Although only in a preliminary study, the effects of rikkunshito on appetite loss in elderly patients with dementia were investigated by Utumi et al. [11]. Rikkunshito was administered for 4 weeks in six elderly patients with dementia suffering from appetite loss in combination with olanzapine + sulpiride, donepezil + paroxetine, trazodone, quetiapine, or donepezil + quetiapine or as monotherapy. In one patient, investigation was stopped because of the development of cholecystitis (administration of rikkunshito was ruled out as being of relevance because of the presence of gallstones and history of cholecystitis). Significant increases in food intake were observed following administration of rikkunshito, with the side effect of mild lower limb edema occurring in two patients.

The ameliorative effects of rikkunshito (1000 mg/kg) in rats administered SSRI (fenfluramine 2 or 5 mg/kg) that had induced gastrointestinal dysmotility were also reported by Fujitsuka et al. [45]. Rikkunshito reversed the disruption of Phase III-like contractions and decreased food intake by restoring the reduced ghrelin secretion via 5-HT_2C_R receptor antagonism. These studies suggest that rikkunshito reduces SSRI-induced adverse events and improves QOL related to gastrointestinal symptoms, without affecting the antidepressant effect of SSRI.

#### 3.3.4. Rikkunshito and Anti-Parkinson Drugs

Effects of rikkunshito on gastroparesis in Parkinson's disease patients were reported by Doi et al. [12]. Twenty patients with mild gastrointestinal symptoms were enrolled; 14 of the 20 patients had constipation. Sixteen patients were taking levodopa/carbidopa, 2 were taking dopamine agonists, and the others were not treated yet. Twelve weeks after rikkunshito administration, 67% of patients reported improvement of their gastrointestinal symptoms, particularly appetite loss and bloating. Rikkunshito significantly shortened the gastric emptying time in these patients measured by the ^13^C-sodium acetate expiration breath test, without any adverse effects, except for its bitter taste.

#### 3.3.5. Rikkunshito and Bioavailability of Antimicrobial Agents

The effects of rikkunshito on the bioavailability of ofloxacin in seven healthy volunteers [13] and that of levofloxacin in eight healthy volunteers [14] were investigated in an open, random crossover study by Hasegawa et al. Subjects were administered a single oral dose of either ofloxacin or levofloxacin alone or by coadministration of rikkunshito at 1-week intervals. No significant differences in any estimated bioavailability parameters of ofloxacin or levofloxacin were observed between the two groups. Urinary recovery of ofloxacin and levofloxacin was not significantly different compared with that after coadministration of rikkunshito.

#### 3.3.6. Rikkunshito and Human Metabolic Enzymes

The effects of rikkunshito on the activity of cytochrome P450 (CYP), a superfamily of drug-metabolizing enzymes, and P-glycoprotein (P-gp), a major drug transporter, were investigated by Ito et al. [60]. The inhibition rate of rikkunshito on human CYP3A4, 2C9, 2C19, 2D6, and 2E1 was less than 50% at concentrations below 0.1 mg/mL. Furthermore, rikkunshito did not affect ATPase activity using human P-gp membranes at concentrations lower than 0.1 mg/mL, in either the presence or absence of P-gp substrate. These findings indicate that rikkunshito is unlikely to cause clinically relevant drug interactions involving the inhibition of major CYP isozymes or P-gp.

## 4. Conclusion

Rikkunshito administration has shown its effects with regard to improvement in the symptoms of GERD and in functional and drug-associated dyspepsia through its effects on upper gastrointestinal functions and ghrelin secretion and signaling. Rikkunshito in combination with anticancer drugs also appeared to ameliorate anorexia, improve QOL, and even prolong survival compared with Western drug monotherapy. Rikkunshito in combination with PPI therapy also showed beneficial effects in PPI-refractory GERD patients and patients with gastrointestinal symptoms after ESD compared with monotherapy. Rikkunshito reduced antidepressant-induced adverse events and improved QOL without affecting antidepressant effects. Rikkunshito showed no significant effect on the bioavailability and renal excretion of antimicrobial agents.

However, because the studies described above were all performed in Japan on account of the nonavailability of standardized rikkunshito outside Japan, the basic and clinical effects of rikkunshito in other countries may not be consistent with these data. In addition, to confirm the safety and efficacy of rikkunshito, multiple, randomized placebo-controlled trials (preferably international) using common endpoints are required. On account of the increasing use of Kampo medicines, accurate data on interactions between these and Western drugs are required, not only for patients but also by healthcare providers.

In conclusion, at present, rikkunshito is considered to have no influence on the efficacy and bioavailability of Western drugs. More importantly, it has shown ameliorative effects on adverse reactions induced by various Western drugs and sometimes yields better results in combination with, for example, anticancer drugs and PPIs than Western drug monotherapy.

## Figures and Tables

**Table 1:** Administration of rikkunshito in combination with Western drugs in human studies.

Reference	Western drugs	Patients	Outcome
[4]	Docetaxel/5-FU/cisplatin	Esophageal cancer	Improved QOL
[5]	S-1/cisplatin	Unresectable or relapsed gastric cancer	Improved anorexia
[6]	Gemcitabine	Stage III/IV pancreatic cancer with ascites	Prolonged median survival
[7]	Rabeprazole	PPI-refractory GERD	Improved GERD score
[8]	Lansoprazole	PPI-refractory LPR	Improved global sensation VAS scores
[9]	Rabeprazole	Gastrointestinal symptoms after ESD	Improved GSRS score
[10]	Fluvoxamine	Depressive disorder	Improved GSRS scores
[11]	Antidepressants or antipsychotics	Elderly patients with dementia	Increase in food intake
[12]	Anti-Parkinson drugs	Parkinson's disease	Ameliorated gastroparesis
[13]	Ofloxacin	Healthy volunteers	No effects on bioavailability
[14]	Levofloxacin	Healthy volunteers	No effects on bioavailability

ESD: endoscopic submucosal dissection; GERD: gastroesophageal reflux disease; LPR: laryngopharyngeal reflux; PPI: proton-pump inhibitor; QOL: quality of life; GSRS: Gastrointestinal Symptom Rating Scale; VAS: visual analog scale.

## Abbreviations

CYP: Cytochrome P450
ESD: Endoscopic submucosal dissection
FD: Functional dyspepsia
GERD: Gastroesophageal reflux disease
GHS-R: Growth hormone secretagogue receptor
GSRS: Gastrointestinal Symptom Rating Scale
LPR: Laryngopharyngeal reflux
P-gp: P-glycoprotein
PPI: Proton-pump inhibitor
QOL: Quality of life
5-HTR: Serotonin receptor
SSRIs: Selective serotonin reuptake inhibitors
VAS: Visual analog scale.
